# Time to Intubation Is Associated with Outcome in Patients with Community-Acquired Pneumonia

**DOI:** 10.1371/journal.pone.0074937

**Published:** 2013-09-19

**Authors:** Sami Hraiech, Julie Alingrin, Stéphanie Dizier, Julie Brunet, Jean-Marie Forel, Bernard La Scola, Antoine Roch, Laurent Papazian, Vanessa Pauly

**Affiliations:** 1 Aix-Marseille Univ, URMITE CNRS-UMR 7278, Marseille, France; APHM, Hôpital Nord, Réanimation, Marseille, France; 2 Direction de la Recherche Clinique, Marseille, France; 3 URMITE, UMR CNRS 7278, L’Institut de Recherche pour le Développement 198, INSERM U1095, Faculté de Médecine, Aix-Marseille Université, Marseille, France; 4 Aix-Marseille Univ, Laboratoire de Santé Publique EA3279, Marseille, France; APHM, Hôpitaux Sud, Service de Santé Publique et d’Information Médicale, Marseille, France; Alberta Provincial Laboratory for Public Health/University of Alberta, Canada

## Abstract

**Introduction:**

It has been suggested that delayed intensive care unit (ICU) transfer is associated with increased mortality for patients with community-acquired pneumonia (CAP). However, ICU admission policies and patient epidemiology vary widely across the world depending on local hospital practices and organizational constraints. We hypothesized that the time from the onset of CAP symptoms to invasive mechanical ventilation could be a relevant prognostic factor.

**Methods:**

One hundred patients with a CAP and necessitating invasive mechanical ventilation were included. Prospectively collected data were retrospectively analysed. Two study groups were identified based on the time of the initiation of invasive mechanical ventilation (rapid respiratory failure requiring mechanical ventilation within 72 h of the onset of CAP and progressive respiratory failure requiring invasive mechanical ventilation 4 or more days after the onset of CAP).

**Results:**

Excepting more COPD patients in the rapid respiratory failure group and more patients with diabetes in the progressive respiratory failure group, these patients had similar characteristics. The overall in-hospital mortality rate was 28% in the rapid respiratory failure group and 51% in the progressive respiratory failure group (*P* = 0.03). The ICU and the day 30 mortality rates were higher in the progressive respiratory failure group (47% vs. 23%, *P* = 0.02; and 37.7% vs. 21.3%, *P* = 0.03; respectively). After adjusting for the propensity score and other potential confounding factors, progressive respiratory failure remained associated with hospital mortality only after 12 days of invasive mechanical ventilation.

**Conclusions:**

This study suggested that the duration or delay in the time to intubation from the onset of CAP symptoms was associated with the outcomes in those patients who ultimately required invasive mechanical ventilation.

## Introduction

Approximately 10 to 20% of hospitalized patients with community-acquired pneumonia (CAP) are admitted to an intensive care unit (ICU) [1], [2], [3], [4], [5], [6], [7]. The mortality for patients requiring ICU supportive care ranges from 20–60% [4], [8], [9], [10], [11]. This wide range suggests huge disparities concerning criteria for ICU admission and/or ICU bed availability discrepancies among hospitals [12].

Recent studies have suggested that delayed ICU transfer is associated with increased mortality for patients with CAP [4], [13], [14], [15], [16]. However, ICU admission policies and patient epidemiology vary widely across the world depending on local hospital practices and organizational constraints, such as bed availability, which is an important confounding factor. The time to hospital admission is also dependent on many factors, such as socio-economic condition, distance from the hospital and clinical stability. In contrast, the right time to start invasive mechanical ventilation varies less among countries due to less subjectivity (clinical judgment of individual physicians). We therefore hypothesized that the time from the onset of CAP symptoms to invasive mechanical ventilation could be a relevant prognostic factor. The main aim of this study was to show that in patients who ultimately required invasive mechanical ventilation, the patients presenting with progressive respiratory failure had a higher risk of death than patients presenting with rapid respiratory failure requiring invasive mechanical ventilation early in the course of their illness.

## Materials and Methods

This study was based on data from a prospective cohort study conducted in the medical ICU of a teaching hospital. One hundred consecutive patients between January 2007 and December 2011 with a diagnosis of CAP requiring invasive mechanical ventilation were included. All patients ≥18 years of age admitted to our medical ICU with the diagnosis of CAP were prospectively identified and monitored until discharge. The study was designed retrospectively but data were prospectively collected. The study was approved by the hospital’s ethical committee (Institut Fédératif de Recherche 48), and the requirement for informed consent was waived because of the observational nature of the study.

### Data Source

Patients with CAP were prospectively identified from a specific electronic questionnaire included in the ICU medical records. All of the data used for this study were therefore prospectively collected.

### Inclusion and Exclusion Criteria

CAP was defined based on the presence of two or more symptoms or signs of CAP [cough (productive or nonproductive), pleuritic chest pain, shortness of breath, temperature >38°C, and crackles or bronchial breath sounds on auscultation] plus radiographic evidence of pneumonia as interpreted by the emergency department physician, pulmonologist or intensivist. CAP onset was defined by the presence of at least two of the following clinical signs: cough (productive or nonproductive), chest pain, shortness of breath, temperature >38°C.

Patients were excluded if they were not invasively ventilated during the ICU stay, if they presented with suspected aspiration pneumonitis (defined as pulmonary opacities in the presence of recent loss of consciousness, vomiting or observation of respiratory distress within 30 min of feeding), tuberculosis, or cystic fibrosis. Patients intubated for more than 12 h or referred from another hospital were also excluded. Patients presenting hospital-acquired pneumonia complicating CAP as the reason for intubation and mechanical ventilation were not taken into account.

### Group Definitions

Two study groups were identified based on the time of the initiation of invasive mechanical ventilation as rapid respiratory failure (requiring mechanical ventilation within 72 h of the onset of CAP) and progressive respiratory failure (requiring intubation 4 or more days after the onset of CAP). This cut off was chosen because it was the median delay between CAP onset and intubation.

### Patient Management

A bronchoalveolar lavage (BAL), 3 sets of blood cultures, and urine samples were obtained on ICU admission. A urine sample was tested for *Legionella pneumophila* serogroup 1 antigen using an enzyme-linked immunosorbent assay. Conventional and molecular methods were used to identify the microorganisms responsible for CAP as previously described [17], [18]. Specific tests included cytomegalovirus, herpes simplex virus, *Mycoplasma pneumoniae*, *Chlamydia pneumoniae*, *Chlamydia psittaci*, *Coxiella burnetii*, *respiratory syncytial virus*, *parainfluenza virus*, *rhinovirus*, *metapneumovirus*, *influenza viruses A and B*, and *adenovirus*.

Initial empiric intravenous antibiotic therapy was ceftriaxone with levofloxacin or erythromycin. For patients who had received a beta-lactam antibiotic during the 90-day period preceding ICU admission, empiric antibiotic therapy with imipenem and levofloxacin was recommended [19]. The antibiotics were started, if not given prior to admission, within the first 2 hours after ICU admission.

All of the patients were ventilated using a lung-protective strategy with a tidal volume of 6 to 8 ml/kg of predicted body weight. Patients presenting acute respiratory distress syndrome (ARDS) with a PaO2: FiO2 ratio<150 were ventilated according to an algorithm that was previously published. [20].

### Data Extraction

The chart review data included demographics, comorbid conditions, physical examination findings, laboratory and microbiology data, and chest radiograph reports. The PIRO (predisposition, insult, response, and organ dysfunction) score [21] was calculated within 24 hours of the ICU admission from 8 variables (presence of chronic obstructive pulmonary disease, immunocompromised state, multilobar opacities on chest radiograph, shock, severe hypoxemia, acute renal failure, bacteraemia and acute respiratory distress syndrome). One point was given for each feature that was present (range, 0–8 points). The 2007 American Thoracic Society/Infectious Disease Society of America (ATS/IDSA) criteria [3] were defined based on the presence of major criteria. The major criteria are a requirement for mechanical ventilation and the presence of septic shock. The patients were classified as having 1 or 2 major ATS/IDSA criteria.

### Diagnostic Criteria

The microbiologic data results were reviewed, and a microbiologic cause was assigned independently by two of the investigators (B.L.S. and L.P.). A patient was considered to have CAP of unknown aetiology if the microbiologic studies were inconclusive.

### Clinical Outcomes

Patients were followed until hospital discharge or death. The primary outcome was in-hospital mortality. The main secondary outcomes were ICU mortality, length of ICU and hospital stays, duration of mechanical ventilation and the number of ventilator-free and survival days.

### Statistical Analysis

The quantitative data are presented as the means and standard deviations (SDs) or medians and interquartile ranges (IQRs) according to the data distribution. The qualitative data are presented as counts (%). A chi-squared test was used to compare categorical variables, and Student’s *t-*test or the Mann-Whitney test was used to compare continuous variables according to the data distribution. A survival analysis was conducted to study the risk factors (mainly progressive respiratory failure) for hospital mortality. We plotted Kaplan-Meier curves of time to death for patients with rapid or progressive respiratory failure.

Because the time to intubation was not randomly assigned in this observational study, we used a propensity score for adjustment. The propensity score corresponded to the conditional probability of invasive mechanical ventilation 4 or more days after the onset of CAP for a patient based on his or her baseline characteristics. To calculate a propensity score for being ventilated 4 or more days after CAP onset, a logistic regression was performed to select some of the following baseline variables: age, gender, Karnosfky score, Knaus chronic health status score [22], McCabe classification [23], and the presence of tobacco use, obesity, malnutrition, COPD, alcoholism, IV drug abuse, stroke, dementia, congestive heart failure, ischemic heart disease, diabetes, liver disease, chronic renal failure, immunosuppression, underlying malignancy, statin use, corticosteroid use prior to hospital admission, asthma and non-invasive mechanical ventilation, (NIV) use prior intubation. The backward stepwise logistic regression finally identified the five factors used to determine the propensity score (age, gender, diabetes, congestive heart failure and COPD). NIV was forced as a factor used to calculate the propensity score. Then, quartiles of the propensity score were used in a multivariate Cox regression model, allowing an estimation of the hazard ratio (HR) for mortality associated with rapid or progressive respiratory failure after adjusting for the propensity score quartile.

Univariate and multivariate Cox regression analyses were performed to determine predictive factors of hospital mortality by estimating crude and adjusted HRs and their 95% confidence intervals (CIs). Theses analyses accounted for the time from intubation to death. The factors with a *P*-value<0.20 in the univariate analysis were entered in the backward stepwise multiple variables Cox model to identify the independent factors associated with the hospital mortality. The collinearity was tested, and the factors introducing collinearity into the multifactorial analysis were removed. We eliminated non-significant terms from the model in a backward approach, until all of the terms remaining in the model were significant at α = 0.05. The eleven variables entered into the model were the delay from clinical presentation to intubation (≤3 days or >3 days), corticosteroid use prior to hospital admission, alcoholism, liver disease, antibiotic administration at the time of intubation, Sequential Organ Failure Assessment (SOFA) score [24] calculated on the day of intubation, blood urea nitrogen on the day of intubation, white blood cell count on the day of intubation, serum glucose on the day of intubation, mean arterial pressure on the day of intubation and the propensity score. To confirm the robustness of the results, a multivariate Cox regression model was done without the propensity score, but including the following factors: the delay from clinical presentation to intubation (≤3 days or >3 days), corticosteroid use prior to hospital admission, alcoholism, liver disease, antibiotic administration at the time of intubation, Sequential Organ Failure Assessment (SOFA) score [24] calculated on the day of intubation, blood urea nitrogen on the day of intubation, white blood cell count on the day of intubation, serum glucose on the day of intubation, mean arterial pressure on the day of intubation, age, gender, serum sodium the day of intubation, multilobar infiltrates the day of intubation and COPD. NIV was forced and introduced in the model.

For both analysis (with and without the propensity score), we assessed the proportional hazard assumption by testing the first-order interaction terms for rapid or progressive respiratory failure with time. All of the statistical analyses were two-sided, and *P*<0.05 was considered to be statistically significant.

All of the statistical analyses were performed using SAS 9.2 software (SAS Institute Inc. Cary, NC).

## Results

During the study period, 1742 patients were admitted to the ICU. A total of 121 patients of the 239 admitted to the ICU with a diagnosis of CAP required invasive mechanical ventilation. Twenty-one of them were excluded, and 100 patients admitted to the ICU with a diagnosis of severe CAP requiring invasive mechanical ventilation were included. Of these, 47% (n = 47) of patients had a rapid respiratory failure, and 53 patients had a progressive respiratory failure.

### Patient Characteristics

Table 1 shows the characteristics for all of the patients and by study group according to the delay separating CAP onset from intubation. Except for more COPD patients in the rapid respiratory failure group and more patients with diabetes in the progressive respiratory failure group, these patients had similar demographic, comorbid, physiologic, laboratory, and radiologic characteristics. In the rapid respiratory failure group, the pH was lower than the progressive respiratory failure group (*P* = 0.02). This more profound acidosis could be related to a slight increase in the PaCO2 observed in the same group. More patients from the progressive respiratory failure group also received antibiotics at the time of intubation. Interestingly, the Simplified Acute Physiology Score (SAPS) II and SOFA scores were similar between the two groups. There was also no difference regarding CAP-specific scores.

**Table 1:** Characteristics of the patients.

	All patientsN = 100	Delay from CAPonset to intubation≤3 days N = 47	Delay from CAPonset to intubation>3 days N = 53	*P* value
**Age**	57±17	56±18	58±17	0.53
**Gender, Male**	69	30 (69)	39 (74)	0.40
**Karnofsky score**	77±20	76±20	79±21	0.44
**Knaus C or D**	45	21 (45)	24 (45)	0.89
**McCabe 1 or 2**	55	28 (60)	27 (51)	0.51
**Direct ICU admission from the community**	53	30 (64)	23 (43)	0.07
**Delay between CAP onset and hospital admission**	2.5±3.2	0.7±0.9	4.2±3.6	0.001
**Delay betweeen CAP onset and ICU admission**	4.0±3.9	1.0±1.1	6.6±3.7	0.001
**Delay betweeen CAP onset and MV**	4.5±4.0	1.3±1.1	7.3±3.6	0.001
**Delay between hospital and ICU admissions**	1.5±2.5	0.4±0.8	2.5±3.1	0.001
**Delay between hospitalization and MV**	2.3±4.0	0.7±0.9	3.7±5.1	0.001
**Delay between ICU admission and MV**	0.5±1.0	0.3±0.6	0.7±1.2	0.06
**Preexisting comorbid conditions and treatments**				
** Obesity**	20	9 (19)	11 (21)	0.96
** Malnutrition**	7	3 (6)	4 (8)	1.0
** Active smoking**	46	21 (45)	25 (47)	0.96
** COPD**	31	20 (43)	11 (21)	0.03
** Asthma**	5	2 (4)	3 (6)	1.0
** Alcoholism**	21	8 (17)	13 (25)	0.50
** IV drug abuse**	7	3 (6)	4 (8)	1.0
** Stroke**	3	3 (6)	0 (0)	0.10
** Dementia**	5	3 (6)	2 (4)	0.66
** Congestive heart failure**	12	8 (17)	4 (8)	0.25
** Ischemic heart disease**	11	7 (15)	4 (8)	0.39
** Diabetes**	18	4 (9)	14 (26)	0.04
** Hepatic insufficiency**	10	5 (11)	5 (9)	1.0
** Chronic renal failure**	4	1 (2)	3 (6)	0.62
** Immunosuppression**	20	9 (19)	11 (21)	0.96
** Underlying malignancy**	15	5 (11)	10 (19)	0.38
** Statins**	13	6 (13)	7 (13)	0.82
** Corticosteroids**	18	10 (21)	8 (15)	0.59
**On ICU admission**				
** SAPS II**	47±19	48±17	45±20	0.44
** SOFA**	8.4±3.5	8.9±3.6	7.9±3.4	0.19
** Receiving corticosteroids**	23	12 (26)	11 (21)	0.74
**NIV prior intubation**	51	25 (53)	26 (49)	0.83
**The day of intubation and mechanical ventilation**				
** Temperature**	38.2±1.3	38.2±1.4	38.3±1.3	0.71
** Heart rate**	110±22	111±24	110±20	0.78
** Mean arterial pressure**	84±17	85±17	83±16	0.56
** pH**	7.29±0.14	7.25±0.15	7.32±0.11	0.02
** PaCO2**	56±20	60±22	53±16	0.10
** PaO2:FiO2 ratio**	124±57	134±53	116±60	0.13
** Blood urea nitrogen**	9.4±7.4	8.9±6.7	9.8±8.0	0.57
** Serum sodium**	141±6	143±4	140±7	0.03
** Serum glucose**	11±4	10±4	11±5	0.76
** White blood cell count**	15±12	13±7	17±15	0.10
** Platelets**	239±140	211±101	263±164	0.06
**Lactates >2 mmol/l**	36	18 (36)	18 (43)	0.65
**ALAT>60 UI/L**	38	24 (53)	36 (68)	0.20
**ASAT>50 UI/L**	57	20 (44)	21 (40)	0.78
**Bilirubinemia>22 µmol/L**	29	15 (32)	14 (26)	0.70
** Multilobar infiltrates**	77	40 (85)	37 (70)	0.07
** Fine IV or V**	84	38 (81)	46 (87)	0.59
** ATS 2 major factors**	85	39 (83)	46 (87)	0.80
** CURB 65≥3**	72	13 (28)	15 (28)	0.88
** PIRO**	3.9±1.2	3.9±1.0	3.8±1.3	0.85
** SOFA**	8.8±3.1	9.1±3.3	8.5±2.9	0.31
**Fluid>30 ml/kg**	55	23 (50)	32 (60)	0.34
** Vasoactive agents**	83	39 (83)	44 (83)	0.79
** Antibiotics at the time of intubation**	76	31 (66)	45 (85)	0.05

SAPS II, Simplified Acute Physiology Score; SOFA, sequential organ failure assessment;

* Mac Cabe score is an index of the severity of the underlying medical condition (Mac Cabe 0: nonfatal underlying disease; Mac Cabe 1 or 2: ultimately or fatal underlying disease).

### Microbial Pathogen Identification and Adequacy of the Initial Antibiotic Treatment

A pathogen was identified in 66 patients. The identification rate was quite similar between the two groups (rapid respiratory failure, 34 of 47 patients [72%]; progressive respiratory failure, 32 of 53 patients [60%]) (Table 2). Gram-positive cocci and gram-negative bacilli each represented 36% of the isolated microorganisms. Finally, viruses were encountered in 23% of the CAP patients. A bacterial-viral coinfection was found in 7 cases (10.6%). Twenty-four of the 34 patients (71%) presenting a culture-positive pneumonia in the rapid respiratory failure group received adequate anti-infectious therapy in the 24-hr period following intubation. There was no difference compared with the progressive respiratory failure group (20 of 32 patients, 63%).

**Table 2:** Microorganisms responsible for CAP.

		All	Delay from CAPonset to intubation≤3 days	Delay from CAP onset to intubation >3 days
**Gram positive cocci**		**27**	**14**	**13**
	*Streptococcus pneumoniae*	10	7	3
	*Methicillin-sensitive Staphylococcus aureus*	10	3	7
	*Methicillin-resistant Staphylococcus aureus*	3	1	2
	*Streptococcus sp.*	3	2	1
	*Enterococcus faecalis*	1	1	0
**Gram positive bacilli**		**1**	**1**	**0**
	*Mycoplasma pneumoniae*	1	1	0
**Gram-negative bacilli**		**27**	**11**	**16**
	*Legionella pneumophila*	6	3	3
	*Haemophilus influenzae*	6	1	5
	*Pseudomonas aeruginosa*	5	2	3
	*Enterobacter sp.*	4	3	1
	*Klebsiella sp.*	3	1	2
	*Serratia marcescens*	1	1	0
	*Morganella morganii*	1	0	1
	*Prevotella oralis*	1	0	1
**Gram-negative cocci**		**2**	**1**	**1**
	*Moraxella catarrhalis*	1	1	0
	*Veillonella atypica*	1	0	1
**Viruses**		**17**	**8**	**9**
	*Influenza A*	9	4	5
	*Herpes simplex*	6	3	3
	*Respiratory syncytial virus*	1	1	0
	*Varicella-zoster virus*	1	0	1
**All**		**74**	**35**	**39**

### Complications

After intubation, 36% of the patients developed acute renal failure requiring renal replacement therapy. Ninety-five percent of the patients presented with septic shock. In 30% of the cases, an inotrope was added to the treatment. In 71% of the cases, pneumonia was responsible for ARDS. Ventilator-associated pneumonia was a complication for 33 patients. A decrease in the SOFA score from day 1 of mechanical ventilation to day 3 was observed in 68 patients (77% of the survivors, 55% of the patients who ultimately died; *P* = 0.02).

### Clinical Outcomes

**Figure 1:**
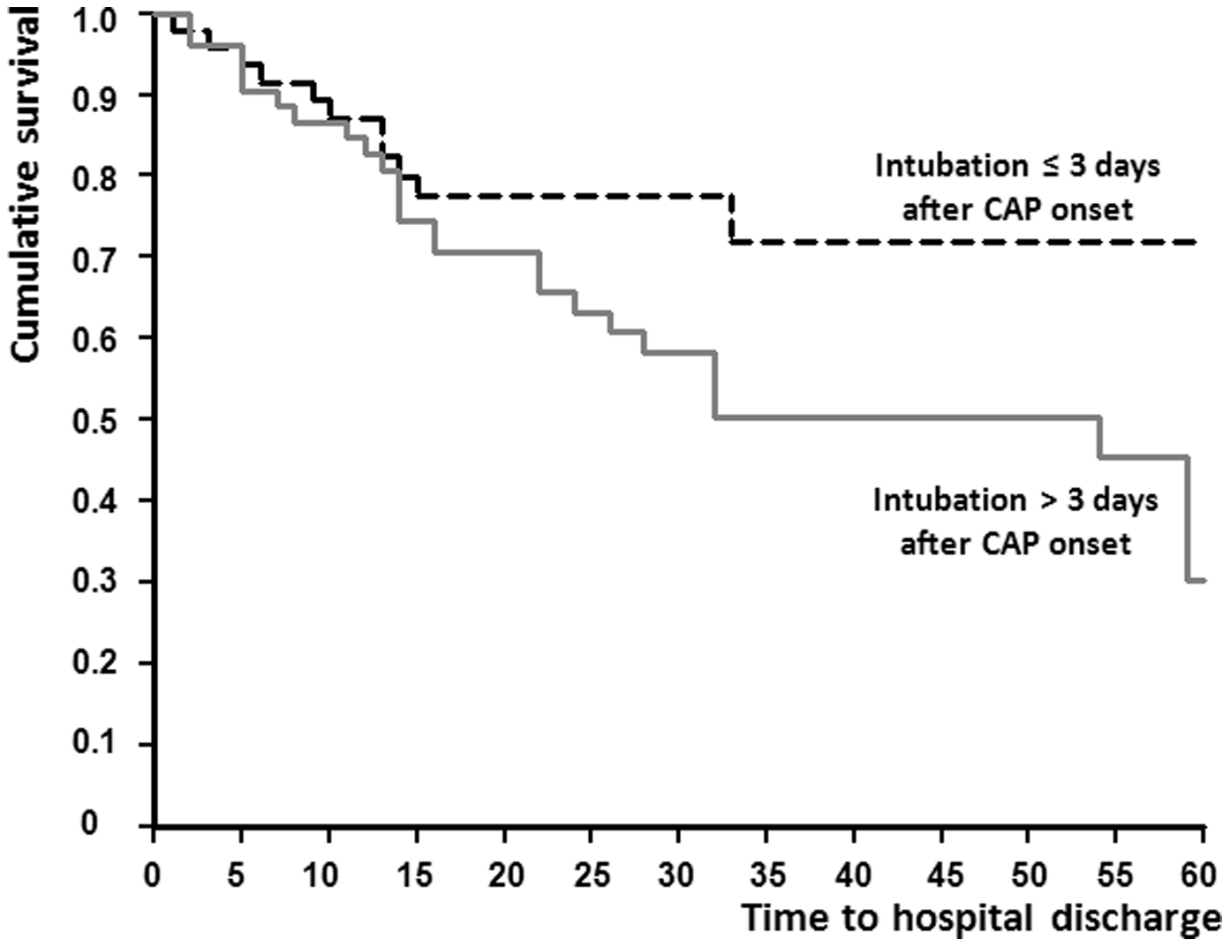
Survival curves from intubation to hospital discharge according to the time elapsed from the onset of CAP to intubation (curves were truncated at day 60 because of the weak number of remaining subjects after day 60).

The overall in-hospital mortality rate was 40%. The mortality rate at day 30 was 30%, whereas the ICU mortality rate was 36%. The overall in-hospital mortality rates was higher in the progressive respiratory failure than the rapid respiratory failure group (51% vs. 28%, *P* = 0.03).The mortality rate at day 30 were 21.3% in the rapid respiratory failure group and 37.7% in the progressive respiratory failure group (*P* = 0.03). The ICU mortality rate was also higher in the progressive respiratory failure than in the rapid respiratory failure group (47% vs. 23%, *P* = 0.02). The in-hospital mortality rate was 41.2% for patients presenting with two major ATS/IDSA criteria. The in-hospital mortality rate was 34% (15 of 44) for patients receiving adequate antibiotic therapy at the time of intubation, 50% (11 of 22) for patients receiving inadequate antibiotic therapy (NS), and 41% (14 of 34) when the microbiological testing remained negative. The duration of mechanical ventilation was not significantly different between the 2 groups (16 days [IQR from 8.5 to 27 days] for the progressive respiratory failure group *vs.* 11 days [IQR from 5 to 22 days] for the rapid respiratory failure group, *P* = 0.15). The median duration of an ICU stay was 21 days (IQR from 11.5 to 31 days) for the progressive respiratory failure group and 14 days (IQR from 8 to 27 days) for the rapid respiratory failure group (*P* = 0.23). The median duration of a hospital stay was 24 days (IQR from 14 to 46 days) for the progressive respiratory failure group and 23 days (IQR from 13 to 44 days) for the rapid respiratory failure group (*P* = 0.77). The number of ventilator-free days at day 28 was higher for patients from the rapid respiratory failure group (10±10 days vs. 5±8 days; p = 0.007). The survival curves are presented in Figure 1 showing that hospital survival was better for patients from the rapid respiratory failure group intubated 3 days or less after CAP onset (log-rank test = 0.02). The univariate analysis showed that more patients in the progressive respiratory failure group died during the hospital stay (Table 3). Among the patients who died, the delay between CAP onset and ICU admission was increased. In contrast, there was no relationship between death and the delay between CAP onset and hospital admission. Interestingly, a direct ICU admission from the community was not related to the outcome. Non-invasive ventilation did not alter the outcome.

**Table 3:** Prognostic factors of hospital mortality.

	Alive at hospitaldischarge N = 60	Dead at hospitaldischarge N = 40	HR (CI95%)	P value
**Age**	54±16	63±17	1.03 (1.01–1.05)	0.008
**Gender, Male**	39 (65)	30 (75)	1.84 (0.86–3.93)	0.11
**Karnofsky score**	80±18	73±22	0.99 (0.97–1.01)	0.20
**Knaus C or D**	24 (40)	21 (52)	1.14 (0.61–2.18)	0.67
**McCabe 1 or 2**	30 (50)	25 (62)	1.02 (0.52–1.97)	0.96
**Direct ICU admission from the community**	36 (60)	17 (42)	0.80 (0.43–1.50)	0.48
**Delay between CAP onset and hospital admission**	2.1±2.6	3.1±3.9	1.09 (1.00–1.20)	0.11
**Delay between CAP onset and ICU admission**	3.1±2.9	5.4±4.8	1.12 (1.04–1.20)	0.002
**Delay between CAP onset and MV**	3.5±3.1	5.9±4.8	1.12 (1.04–1.20)	0.003
**Delay between CAP onset and MV>3 days**	26 (43)	27 (67)	2.24 (1.11–4.55)	0.025
**Delay between hospital admission and MV**	2.0±4.5	2.8±3.2	1.02(0.96–1.09)	0.47
**Delay between hospital admission and ICU**	1.0±2.0	2.3±3.0	1.1 (1.01–1.2)	0.03
**Delay between ICU admission and MV**	0.5±0.9	0.5±1.0	0.80(0.71–1.31)	0.96
**Obesity**	12 (20)	8 (20)	1.10 (0.51–2.40)	0.81
**Malnutrition**	3 (5)	4 (10)	0.80(0.38–3.09)	0.43
**Active smoking**	26 (43)	20 (50)	1.40 (0.74–2.64)	0.30
**COPD**	19 (32)	12 (30)	0.84 (0.43–1.67)	0.63
**Asthma**	4 (7)	1 (2)	0.70(0.96–5.12)	0.64
**Alcoholism**	9 (15)	12 (30)	1.76 (0.88–3.51)	0.11
**IV drug abuse**	6 (10)	1 (2)	0.29 (0.04–2.14)	0.23
**Stroke**	3 (5)	0 (0)	0.05 (0.01–114)	0.44
**Dementia**	5 (8)	0 (0)	0.05 (0.01–82)	0.42
**Congestive heart failure**	6 (10)	6 (15)	1.22 (0.51–2.92)	0.65
**Ischemic heart disease**	6 (10)	5 (12)	1.28 (0.50–3.30)	0.61
**Diabetes**	8 (13)	10 (25)	1.71 (0.82–3.53)	0.15
**Hepatic insufficiency**	4 (7)	6 (5)	2.50 (1.04–6.01)	0.04
**Chronic renal failure**	2 (3)	2 (5)	2.25 (0.54–9.43)	0.27
**Immunosuppression**	10 (17)	10 (25)	1.05 (0.50–2.17)	0.91
**Underlying malignancy**	7 (12)	8 (20)	1.32 (0.60–2.91)	0.49
**Statins**	9 (15)	4 (10)	0.66 (0.24–1.87)	0.44
**Corticosteroids prior hospital admission**	11 (18)	7 (17)	0.78 (0.34–1.79)	0.55
**SAPS II on ICU admission**	41±15	55±20	1.04 (1.02–1.06)	0.0001
**SOFA on ICU admission**	7.5±2.9	9.7±4.0	1.17(1.08–1.27)	<0.001
**NIV prior intubation**	29 (48)	22 (55)	1.03 (0.55–1.93)	0.92
**Temperature** *	38.3±1.4	38.1±1.3	0.98(0.78–1.22)	0.84
**Heart rate** *	112±23	108±20	0.99 (0.98–1.01)	0.85
**Mean arterial pressure** *	86±18	80±13	0.98 (0.96–1.00)	0.052
**pH** *	7.30±0.12	7.27±0.16	0.61 (0.05–7.21)	0.70
**PaCO2** *	55±20	58±19	1.00 (0.98–1.02)	0.97
**PaO2:FiO2 ratio** *	126±54	122±61	1.00 (0.99–1.01)	0.68
**Blood urea nitrogen** *	8.5±8.1	10.7±6.0	1.04(1.00–1.07)	0.029
**Serum sodium** *	141±6	142±6	1.01 (0.95–1.07)	0.78
**Serum glucose** *	9±2	12±6	1.08 (1.01–1.15)	0.019
**White blood cell count** *	14±8	18±17	1.04(0.99–1.04)	0.08
**Platelets** *	240±114	237±173	0.99 (0.99–1.01)	0.47
**Lactates>2 mmol/l** *	18 (33)	18 (49)	1.92 (1.01–3.68)	0.048
**ALAT>60 UI/L** *	22 (37)	16 (41)	1.73 (0.90–3.34)	0.10
**ASAT>50 UI/L** *	32 (54)	25 (64)	1.55 (0.80–2.99)	0.20
**Bilirubinemia>22 µmol/L** *	15 (25)	14 (35)	1.84 (0.95–3.55)	0.07
**Multilobar infiltrates** *	47 (78)	30 (75)	0.79 (0.39–1.64)	0.53
**Fine IV or V on ICU admission**	48 (80)	36 (90)	1.76 (0.63–4.97)	0.28
**ATS 2 major factors on ICU admission**	50 (83)	35 (87)	0.99 (0.39–2.56)	0.99
**CURB 65≥3 on ICU admission**	36 (60)	36 (90)	6.4 (1.84–22)	0.004
**PIRO** *	3.6±1.2	4.3±1.0	1.35 (1.04–1.75)	0.025
SOFA**	8.0±2.4	10.0±3.6	1.19 (1.09–1.30)	0.0001
**Fluid>30 ml/kg** *	29 (48)	16 (40)	0.74 (0.38–1.41)	0.35
**Vasoactive agents** *	47 (78)	36 (90)	2.26 (0.79–6.38)	0.13
**Antibiotics at the time of intubation**	42 (70)	34 (85)	2.08 (0.87–4.98)	0.10

* the day of intubation and mechanical ventilation; CI 95%, 95% confidence interval for Hazard ratio; SAPS II, Simplified Acute Physiology Score; SOFA, sequential organ failure assessment; *Mac Cabe score is an index of the severity of the underlying medical condition (Mac Cabe 0: nonfatal underlying disease; Mac Cabe 1 or 2: ultimately or fatal underlying disease).

In the Cox proportional hazard model after adjusting for the propensity score (quartiles) and other potential confounding factors, progressive respiratory failure remained associated with in-hospital mortality only after 12 days of mechanical ventilation. Indeed, we tested the proportional hazard assumption for the factor “delay from clinical presentation to intubation” for the interaction between this factor and time. This interaction was significant (*P = *0.02). Consequently, the cut off value for time was 12 days of mechanical ventilation (time point where the two survival curves begin to differ) to estimate the effect of the factor “delay from clinical presentation to intubation” on hospital mortality before and after 12 days (Table 4). The Cox analysis performed without taking into account the propensity score confirmed that progressive respiratory failure was associated with in-hospital mortality (after 10 days, data not shown).

**Table 4:** Independent risk factors of hospital death.

	Hazard ratio	CI95% for HR	P value
**Hepatic insufficiency**	3.0	1.2 to 9.0	0.03
**SOFA score** *	1.2	1.1 to 1.3	<0.0001
**Delay between CAP onset and MV>3 days**			
**- Mortality within the first 11 days**	1.2	0.4 to 3.7	0.7
** - Mortality at day 12 or more**	3.4	1.2 to 10.8	0.026

CI 95%, 95% confidence interval for Hazard ratio; SOFA, sequential organ failure assessment;

* ,The day of intubation and beginning of invasive mechanical ventilation.

Backward multivariate Cox regression analysis adjusted using a propensity score (quartiles). Variables entered into the model: propensity score, alcoholism, receiving corticosteroids at the time of intubation, receiving antibiotics at the time of intubation; the day of intubation: blood urea nitrogen, serum glucose, mean arterial pressure, white blood cell count, SOFA score; and a delay between CAP onset and mechanical ventilation >3 days.

## Discussion

This study showed that the time to intubation from the onset of CAP symptoms was associated with the outcome in patients who ultimately required invasive mechanical ventilation. The longer the delay, the higher was the in-hospital mortality. This result strongly suggests that in the most severe cases of CAP (those requiring invasive mechanical ventilation), a rapid worsening of the respiratory status maybe associated with faster patient management (rapid transfer from home to the hospital, direct transfer from the ED to the ICU, rapid identification of respiratory failure in the intermediate care units). Conversely, the patients presenting progressive respiratory failure are admitted later to the ICU. On ICU admission and the day of intubation, these patients did not exhibit significant differences compared with the patients admitted earlier.

Very likely, patients presenting with rapid respiratory failure received more aggressive treatment and were promptly admitted to the ICU where sophisticated diagnostic and therapeutic methods were administered. In the present study, ICU mortality prediction methods, such as the SAPS II [25] and SOFA, had good predictive characteristics for hospital outcome, similar to when the CURB-65 [26] was ≥3 on ICU admission and for the PIRO score calculated on the day of intubation.

In the present study, direct admission to the ICU was not related to the prognosis. This factor has been extensively reported as being strongly associated with the outcome of patients presenting CAP [1], [4], [8], [14], [15], [27], [28], [29], [30], [31], [32]. However, the ICU admission rates and policies vary markedly among health care systems and depend on a number of factors other than pneumonia severity. While patients who require emergent mechanical ventilation and/or vasopressors (defined by the IDSA/ATS as major criteria for severe CAP) and hence direct ICU admission may easily be identified, a clinical picture at home or in the ED that suggests rapidly progressive pneumonia is much more difficult to identify, especially for a non-intensivist. In many places, the decision to admit a patient to the ICU is related to the need for mechanical ventilation or vasopressor support. [1], [3], [11], [13], [21], [33], [34]. Severity illness scores have been suggested to be helpful at the time of hospital admission (mainly for the ED) because CAP is an erratically evolving process during the first few days. Moreover, as discussed above, there is great variability concerning the ICU admission criteria from one hospital to another and from one country to another. The onset of CAP symptoms is generally easy to determine. The criteria to intubate the patients are more objective than those used to admit a patient in an ICU, which is why we took the time elapsed from symptom onset to intubation as the main parameter to study. Possible delayed invasive mechanical ventilation (at home, in the ED, in the ward or in the ICU) is a clinical and organizational problem. As the main parameter, we did not choose the time between hospital admission and intubation because some patients are intubated outside the hospital prior to hospital admission. Interestingly, the delay between hospital admission (or ICU admission) and intubation was not associated with death in the present study, strongly suggesting that pre-hospital factors, such as the role of the general practitioner and social isolation of some patients, are also important to consider to improve the outcome. Moreover, the reluctance to use invasive mechanical ventilation (inside and outside of the ICU) because of the risk of ventilator-associated pneumonia and/or ventilator-induced lung injury can avoid intubation in some cases but also delay intubation in some cases with uncertain impacts on outcome. Finally, the decision to intubate and ventilate a patient presenting with CAP is multifactorial and, in some cases, very subjective. Thus, we chose to study the delay between symptom onset and intubation, which is, from our point of view, much more objective for determining whether progressive respiratory failure worsens the prognosis more than the time passed before admitting the patient to the ICU. The role of non-invasive mechanical ventilation is also unclear. While in some cases, this mode of ventilation most likely avoids tracheal intubation and improves the outcome, in other cases, when the patients are ultimately intubated and ventilated, the prognosis could be worse [35]. In a recent study, Carrillo et al. [36] reported a higher mortality rate when intubation was delayed in a group of patients presenting with respiratory failure related to CAP treated using NIV. In the present study, we did not find any deleterious effect on prognosis using non-invasive mechanical ventilation prior to intubation. However, we did not assess the duration of NIV prior to intubation. Moreover many patients benefited from NIV and were not subsequently invasively ventilated and not enrolled in the present study. It could be very useful in future studies to evaluate if time to intubation is associated with the outcome in specific patients populations such as COPD patients or patients with comorbidities such as diabetes.

Despite the fact that many patients received antibiotics at the time of intubation, the microorganisms suspected to be responsible for the CAP were identified in two-thirds of the patients in the present study using extensive microbiological investigations. Interestingly, there were no differences between the two groups (early and late intubation) regarding the rate of microbial identification. In contrast, mortality was slightly (but not significantly) higher when the patients received adequate antibiotic therapy at the time of intubation. A larger sample size could be useful to specifically evaluate this important point [37].

Statin use prior to ICU admission was prospectively assessed. In contrast to previous reports [38], we did not find any outcome improvement with the use of statins. However, in a recent study showing the beneficial effect of statins [38], only 10.2% of the patients were ventilated or received vasopressors. Moreover, the use of statins was not associated with less mechanical ventilation requirements suggesting that the beneficial effect on prognosis was not related to a decreased need for ventilator support [38].

Our study has limitations that are important to acknowledge. First, this study was designed retrospectively using prospectively collected data. This study was performed in only one ICU at a teaching hospital, raising a concern for the generalizability of our results. However, this fact may also be a strength because many confounding factors regarding prognosis were controlled (antibiotic policy, mechanical ventilation strategy, microbiological exams). The fact that the patient or his/her family self-identifies the onset of CAP symptoms may be a source of error that we have to acknowledge. Finally, in the present series, the ICU mortality rate for patients with community-acquired pneumonia requiring mechanical ventilation was 36%, which is in the lower range of previously reported results [3], [39], [40].

It could be important now to identify what factors might actually result in earlier intervention to design interventional trials with the objective to show a decreased mortality rate related to severe CAP.

In conclusion, our results suggest that time to intubation should be carefully evaluated when there is progressive respiratory failure in patients presenting with symptoms of pneumonia for several days. Further studies are needed to investigate whether admittance of all CAP patients not needing urgent invasive mechanical ventilation or vasopressors to intermediate care units to better assess the need for invasive mechanical ventilation is associated with a better outcome.
